# Endoscopic or Conservative Management of Iatrogenic Duodenal Perforations Caused by Long Plastic Biliary Stent Distal Migration

**DOI:** 10.14309/crj.0000000000000430

**Published:** 2020-07-30

**Authors:** Namisha Thapa, Michael Loudin, Kaveh Sharzehi, Silvio W. de Melo, Brintha K. Enestvedt

**Affiliations:** 1 Department of Medicine, Division of Gastroenterology and Hepatology, Oregon Health & Science University, Portland, Oregon

## Abstract

Plastic biliary stents are associated with rare but potentially life-threatening distal stent migration. We present 4 patient cases with distal migration, whereas the proximal aspect remained in the bile duct. Time to stent migration ranged from 1 week to 2 months. Stent migration caused contralateral duodenal wall perforation; 2 underwent endoscopic over-the-scope clip placement for defect closure. All required previous stent removal and stent exchange. This case series highlights that proximal stricture and longer stents have higher migration risk, also shown in the literature. We also show that duodenal perforation can successfully be managed endoscopically with an over-the-scope clip.

## INTRODUCTION

Biliary stents are used for internal drainage of malignant and benign biliary obstruction during endoscopic retrograde cholangiopancreatography (ERCP). Intestinal perforation is a rare but potentially life-threatening adverse event associated with plastic biliary stents and has been described with distal stent migration out of the biliary tree, occurring in less than 6% of all plastic biliary stents with less than 1% actually causing intestinal perforation.^1,2^ Endoscopic management of iatrogenic duodenal perforations with endoscopic clips to close the defect is gaining momentum.^3^ We present a case series of 4 patients with biliary stricture and duodenal perforation from long in situ plastic biliary stents, managed conservatively or with endoscopic management.

## CASE REPORT

### Patient 1

**Figure 1.**
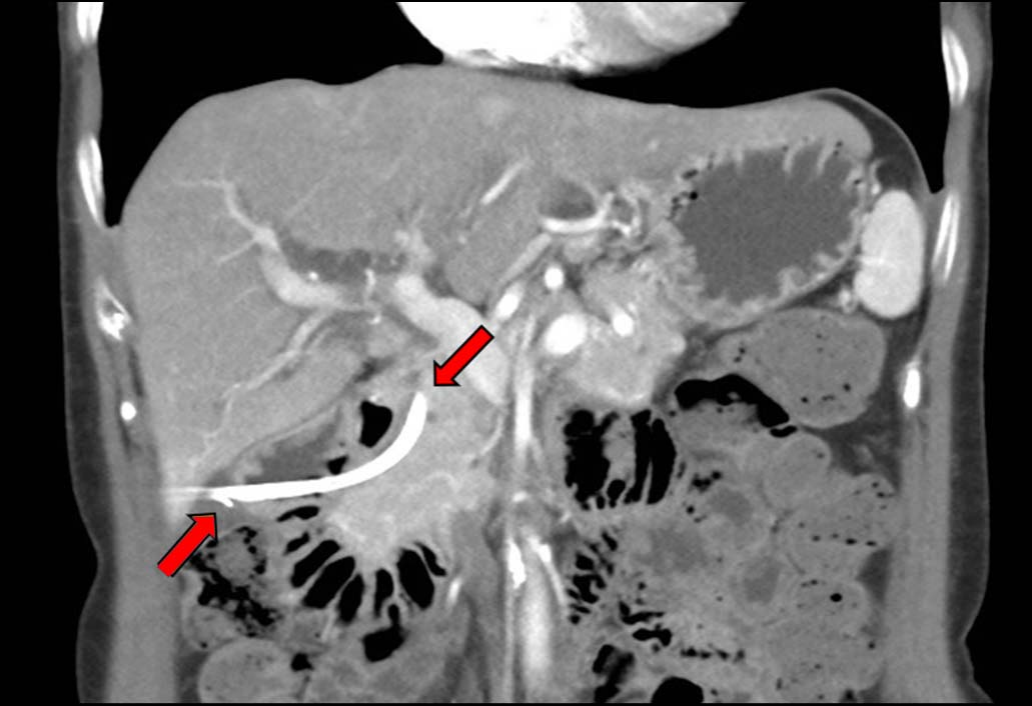
Abdominal computed tomography scan showing duodenal perforation from the distal tip of the biliary stent, whereas its proximal aspect is still within the common bile duct. Red arrows indicate proximal and distal ends of the stent.

**Figure 2.**
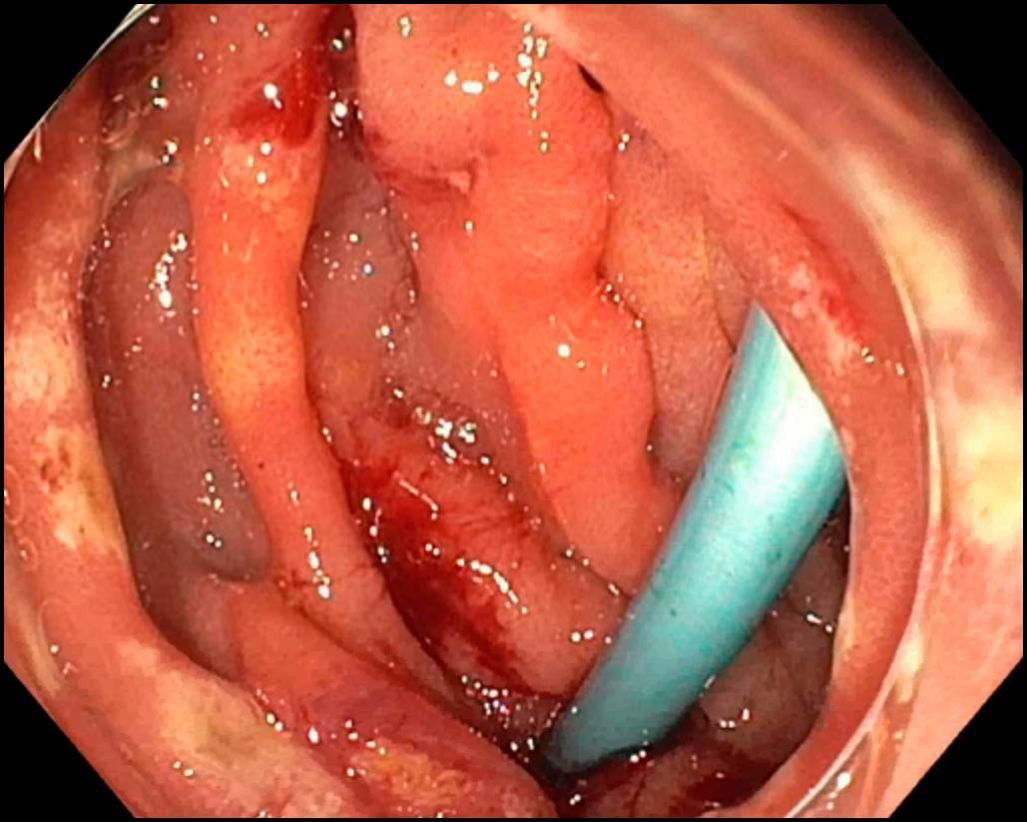
Endoscopic retrograde cholangiopancreatography reveals stent perforation through the wall of the duodenum opposite to the papilla.

**Figure 3.**
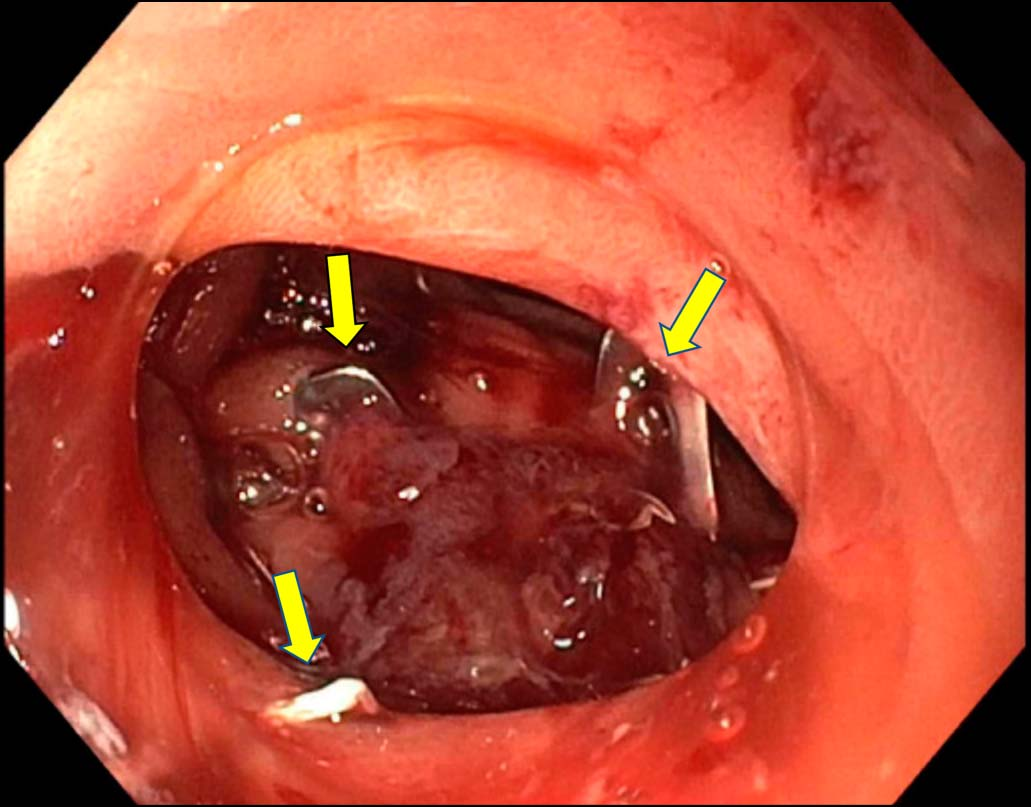
Endoscopic closure of duodenal perforation with an over-the-scope clip (12 × 6 mm GC; OVESCO endoscopy AG). Arrows indicate metal ends of clip placed.

A 65-year-old woman with a history of common hepatic duct cholangiocarcinoma undergoing treatment with gemcitabine and cisplatin who had an indwelling 10 Fr × 12 cm (Cotton-Leung Biliary Stent [CLSO]-10-12, Cook Medical, Winston-Salem, NC) plastic biliary stent into her left hepatic duct placed 2 months earlier presented with severe right upper quadrant (RUQ) pain. She was tachycardic with elevated liver function tests (LFT): aspartate aminotransferase (AST) of 154 IU/L, alanine aminotransferase (ALT) of 280 IU/L, alkaline phosphatase (ALP) of 246 IU/L, and normal total bilirubin at 0.9 mg/dL. Abdominal computed tomography showed duodenal perforation from the distal tip of the biliary stent with its proximal aspect remaining within the common bile duct (Figure 1). Urgent ERCP revealed the stent penetrating through the opposite duodenal wall from the papilla (Figure 2). The plastic stent was removed with rat-tooth forceps, revealing a small focal duodenal perforation. The adjacent mucosa was marked with a biopsy forceps bite to facilitate identification because the fistula was anticipated to close quickly, making finding it challenging. The duodenal perforation was closed with an over-the-scope clip (12 mm × 6 mm GC; Ovesco Endoscopy AG, Germany) with symptom resolution immediately postprocedure (Figure 3). ERCP was completed, and a modified 10 Fr × 10 cm plastic stent with a full external pigtail and a ½ internal pigtail (6108, Hobbs Medical, Stafford Springs, CT) was advanced across the stricture. At a 1-year follow-up, the patient has since needed bilateral metal stents and an enteral stent for the management of her advancing cholangiocarcinoma. Her over-the-scope clip has since spontaneously migrated.

### Patient 2

**Figure 4.**
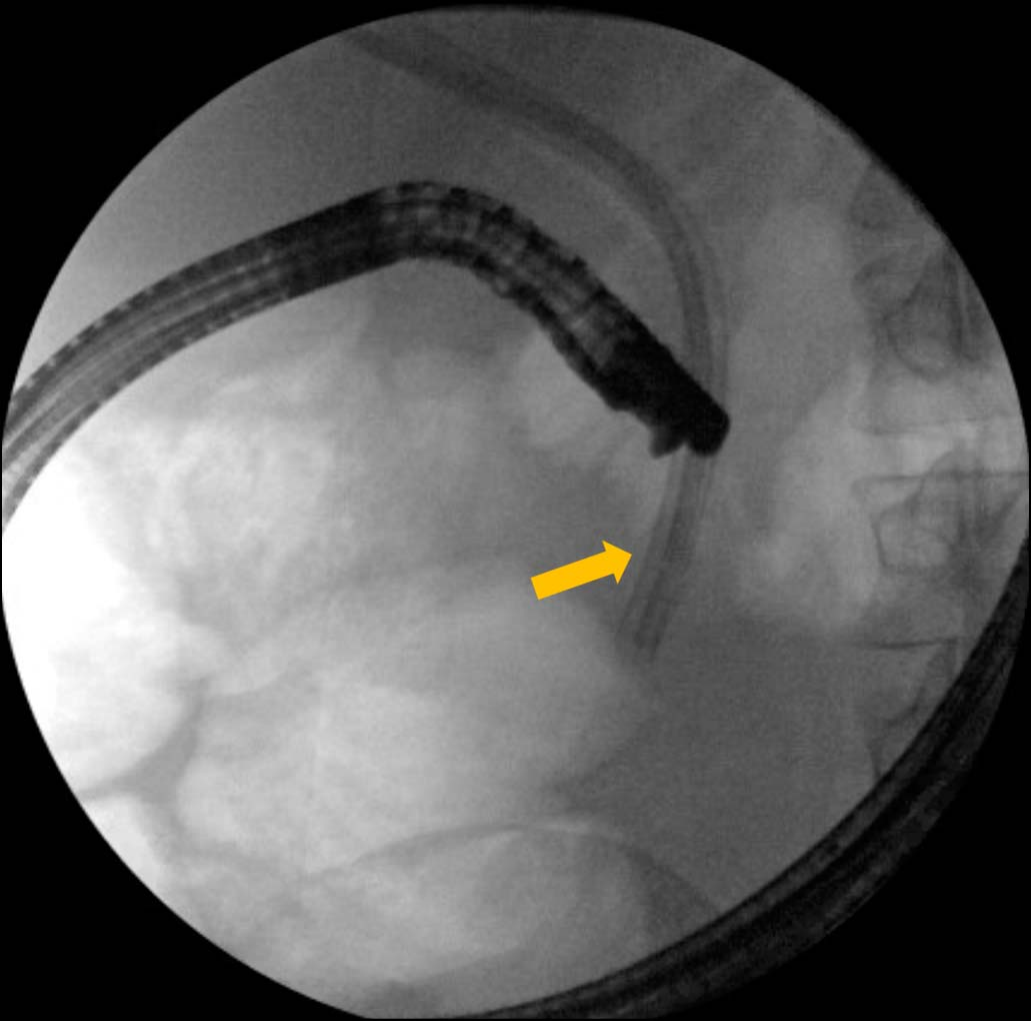
Fluoroscopic image during endoscopic retrograde cholangiopancreatography reveals stents traversing the duodenal wall (arrow is where stent traverses opposing duodenal wall).

A 28-year-old man with a history of orthotopic liver transplant for nonalcoholic steatohepatitis cirrhosis complicated by anastomotic biliary stricture who had been undergoing serial ERCPs for endoscopic therapy to the stricture presented for early biliary stent exchange because of new elevation in LFTs. During ERCP, 3 plastic biliary stents were seen in the second part of the duodenum with deep wall penetration and at least 1 stent suspected of perforating the wall contralateral to the major papilla (Figure 4). The three 10 Fr × 12 cm plastic (CLSO 10-12, Cook Medical) stents were removed using rat-toothed forceps, revealing a suspected fistula at the site of perforation. Biopsy forceps were used to mark the adjacent mucosa for future identification. The perforation was successfully closed with an over-the-scope clip (12 × 6 mm GC, Ovesco Endoscopy AG). ERCP was performed, and a plastic 10 Fr × 10 cm biliary stent with a full external pigtail and a ¾ internal pigtail (6108, Hobbs Medical) was placed into the left hepatic duct. The patient had no symptoms of perforation pre- or post-ERCP and progressed well with diet advancement and discharge on postprocedure day 2. During routine ERCP 2 months later, the over-the-scope clip was still in place, but the biliary stent had partially migrated out of the ampulla. This required a repeat stent exchange with 2 modified 10 Fr × 10 cm biliary stents with a full external pigtail and ¾ internal pigtail (6108, Hobbs Medical). At his 6-month follow-up ERCP, the over-the-scope clip notably was not present and presumably spontaneously dislodged and migrated.

### Patient 3

A 60-year-old man with a history of metastatic colorectal carcinoma to the liver with a hepatic arterial infusion pump-associated biliary sclerosis and placement of a 10 Fr × 12 cm plastic biliary stent (CLSO 10-12, Cook Medical) into the common bile duct 1 month earlier presented with acute RUQ abdominal pain, chills, and fatigue. He was found to have mild leukocytosis, RUQ tenderness, and elevated LFTs (AST of 324 IU/L, ALT of 425 IU/L, and total bilirubin of 6.4 mg/dL) higher from her chronically elevated baseline (AST and ALT in the 50 s IU/L and total bilirubin 1.5 mg/dL). ERCP revealed the biliary stent's tip buried into the mucosa of opposing duodenal wall, causing ulceration without definitive evidence of perforation, however, with suspected deep penetration injury. A rat-toothed forceps was used to remove the stent from the bile duct, and it was replaced with a modified 10 Fr × 10 cm plastic biliary stent with full external pigtail and ¾ internal pigtail (6108, Hobbs Medical). No endoscopic therapy was needed for the duodenal wall injury. He was found to have central hepatic abscess on magnetic resonance imaging, which was successfully drained; he continued to clinically improve, tolerated diet advancement, and was discharged on oral levofloxacin and metronidazole. The patient was readmitted 1 month later with stent occlusion requiring repeat ERCP with stent exchange. At the 8-month follow-up, he had already undergone 4 additional ERCPs for biliary obstruction.

### Patient 4

A 69-year-old man with a history of plastic biliary stent placement for possible autoimmune vs sarcoidosis-related biliary stricture with pathology negative for malignancy a week earlier presented with sudden-onset acute RUQ abdominal pain. He had been on oral prednisone for the presumed diagnoses. He presented with tachycardia, mild hypotension, and RUQ tenderness with guarding. His laboratory test results revealed mild leukocytosis and normal LFTs. Abdominal computed tomography showed the common bile duct stent tip perforating the duodenal wall and free fluid. During ERCP, a plastic biliary stent was seen in the ampulla with its tip extending into the lumen of the duodenum; repeated duodenal evaluation showed no obvious perforation, although a sealed perforation was suspected; no endoscopic therapy was required. The previously placed 10 Fr × 10 cm stent (CLSO 10-10, Cook Medical) was exchanged for a modified 10 Fr × 10 cm plastic biliary stent with a full external pigtail and a ¾ internal pigtail (6108, Hobbs Medical). The patient was admitted to medical intensive care unit postprocedure for septic shock and briefly required norepinephrine in addition to intravenous antibiotics and stress dose steroids. He clinically improved within 24 hours and was transitioned to full liquid diet 2 days before discharge. He was discharged home on a 14-day course of oral ciprofloxacin and metronidazole and continued oral steroids. On a 3-month follow-up, the patient was deemed a poor surgical candidate and underwent ERCP with a plan for fully covered self-expanding metal stent placement, but his stricture had resolved, confirming the diagnosis of autoimmune cholangiopathy. On an magnetic resonance cholangiopancreatography a month later, improved but persistent intrahepatic biliary duct dilatation was noted.

## DISCUSSION

We discuss 4 biliary stricture cases in which patients sustained a duodenal perforation related to partial distal migration of long plastic biliary stents. In this present case series, the time frame between stent placement and reported adverse events ranged from 7 days to 3 months. In previous literature, the time frame has varied from 2 weeks to 3 years with majority ranging from 1 to 6 months.^4^ In all our cases, the stents were either 10 or 12 cm long, were all from the same manufacturer, and the strictures were proximal common hepatic duct or hilar strictures.^5^ In addition, in all our cases, the removed stents were exchanged immediately for double pigtail biliary stents to limit any duodenal damage from the distal aspect of the stent.

As has been documented previously, proximal strictures and longer stents are more prone to stent migration; our case series supports this.^5^ We suspect that the dynamics of a tight proximal stricture plays a role in stent position and potential for migration. In 2 cases of perforation (patients 1 and 2), an over-the-scope clip was successfully placed endoscopically to close the perforation without any clinical consequence or need for escalation of care. We elected to mark the site of duodenal fistula before removal of the penetrating stents, given the previous experience that these perforations are small and close rapidly, thereby making identification of the fistula challenging on subsequent scope insertion for therapy. In the other 2 cases, the patients were managed conservatively without endoscopic therapy, suggesting that these injuries are small, close quickly, have an overall excellent prognosis (possibly related to their retroperitoneal location), and most often can be safely managed endoscopically or with antibiotics alone. With the increasing use of the over-the-scope clip for perforation closure, we anticipate seeing more studies documenting its use for more reliable data (Table 1).

**Table 1.**
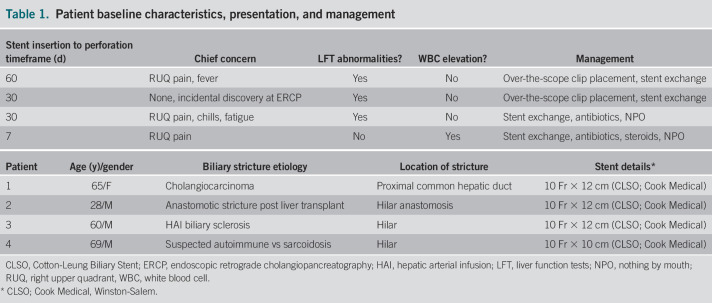
Patient baseline characteristics, presentation, and management

All cases occurred within an 8-month time frame, resulting in an internal review of our stent utilization. Six months earlier, our institution had elected to change to a more cost-effective biliary stent (from Cotton-Huibregtse Biliary Stent to CLSO, Cook Medical, Winston-Salem, NC). A recent case series of 5 patients also noted duodenal perforations in 4 of their 5 patients related to this particular stent type and with lengths 10–12 cm, very similar to our series.^3^ In the years before our switch, we had zero perforations. We transitioned back to Cotton-Huibregtse Biliary Stent, which had a more exaggerated flexion of the distal stent in the duodenum and have encountered no further instances of perforation in the last 8 months.

## DISCLOSURES

Author contributions: N. Thapa wrote the manuscript and reviewed the literature. M. Loudin, K Sharzehi, and SW de Melo edited the manuscript, reviewed the literature, and approved the final version. BK Enestvedt wrote the manuscript, reviewed the literature, approved the final version, and is the article guarantor.

Financial disclosure: None to report.

Previous presentation: This case was presented at the American College of Gastroenterology Annual Scientific Meeting; October 25-30, 2019; San Antonio, Texas.

Informed consent was obtained for this case report.

## References

[1] Chandrasekhara V Khashab MA Muthusamy VR Adverse events associated with ERCP. Gastrointest Endosc. 2017;85(1):32–47. 27546389 10.1016/j.gie.2016.06.051

[2] Yaprak M Mesci A Colak T Yildirim B Biliary stent migration with duodenal perforation. Eurasian J Med. 2008;40(3):154–6. 25610053 PMC4261672

[3] Kim HS Moon HJ Lee NY Endoscopic management of duodenal perforations caused by migrated biliary plastic stents. Endosc Int open. 2019;7(6):E792–5. 31198841 10.1055/a-0887-4200 PMC6561770

[4] Namdar T Raffel AM Topp SA Complications and treatment of migrated biliary endoprostheses: A review of the literature. World J Gastroenterol. 2007;13(40):5397–9. 17879415 10.3748/wjg.v13.i40.5397 PMC4171335

[5] Arhan M Odemis B Parlak E Ertugrul I Basar O Migration of biliary plastic stents: Experience of a tertiary center. Surg Endosc. 2009;23(4):769–75. 18649099 10.1007/s00464-008-0067-x

